# Functional network dynamics in a neurodevelopmental disorder of known genetic origin

**DOI:** 10.1002/hbm.24820

**Published:** 2019-10-22

**Authors:** Erin Hawkins, Danyal Akarca, Mengya Zhang, Diandra Brkić, Mark Woolrich, Kate Baker, Duncan Astle

**Affiliations:** ^1^ MRC Cognition and Brain Sciences Unit University of Cambridge Cambridge UK; ^2^ Oxford Centre for Human Brain Activity University of Oxford, University Department of Psychiatry, Warneford Hospital Oxford UK; ^3^ Department of Medical Genetics University of Cambridge, Cambridge Institute for Medical Research Cambridge UK

**Keywords:** atypical brain development, cognitive development, functional connectivity, human genetics, magnetoencephalography

## Abstract

Dynamic connectivity in functional brain networks is a fundamental aspect of cognitive development, but we have little understanding of the mechanisms driving variability in these networks. Genes are likely to influence the emergence of fast network connectivity via their regulation of neuronal processes, but novel methods to capture these rapid dynamics have rarely been used in genetic populations. The current study redressed this by investigating brain network dynamics in a neurodevelopmental disorder of known genetic origin, by comparing individuals with a *ZDHHC9*‐associated intellectual disability to individuals with no known impairment. We characterised transient network dynamics using a Hidden Markov Model (HMM) on magnetoencephalography (MEG) data, at rest and during auditory oddball stimulation. The HMM is a data‐driven method that captures rapid patterns of coordinated brain activity recurring over time. Resting‐state network dynamics distinguished the groups, with *ZDHHC9* participants showing longer state activation and, crucially, *ZDHHC9* gene expression levels predicted the group differences in dynamic connectivity across networks. In contrast, network dynamics during auditory oddball stimulation did not show this association. We demonstrate a link between regional gene expression and brain network dynamics, and present the new application of a powerful method for understanding the neural mechanisms linking genetic variation to cognitive difficulties.

## 1 INTRODUCTION

In recent years, whole‐brain imaging methods have advanced our ability to characterise functional brain connectivity, and have revealed that distributed neural systems support cognition and behaviour (Astle, Barnes, Baker, Colclough, & Woolrich, 2015; Barnes, Woolrich, Baker, Colclough, & Astle, 2016; Smith et al., 2015; Vidaurre et al., 2017). Disruptions to functional connectivity are considered a characteristic feature of multiple developmental disorders, but surprisingly little is known about the mechanisms that drive this variability. It is well known that genetic factors influence the intrinsic organisation of functional networks (e.g., Bathelt, Astle, Barnes, Raymond, & Baker, 2016, 2017; Colclough et al., 2017; Wang et al., 2015). In many cases, genes are likely to influence the developmental emergence of functional networks via regulation of continuous activity‐dependent physiological processes, rather than by fixed anatomical differences. However, understanding of the rapid synchronisation and integration of functional connectivity networks at fast time‐scales, in the range of 100–200 milliseconds, is limited. This is partly because there is a scarcity of methods capable of characterising the fast dynamics of brain networks. As a result, there is currently little understanding of the genetic effects on these networks and the cellular mechanisms that could drive their developmental variability or the consequences of perturbations to these network dynamics for cognition. The aim of this study is to redress this by exploring dynamic transient brain connectivity in a group of individuals with a neurodevelopmental disorder of known genetic origin.

The current methods capable of deriving system‐wide neural networks largely assume that the networks remain stationary over time. Functional magnetic resonance imaging (fMRI) has been the primary method for investigating coordination between brain regions, by measuring covariations in the haemodynamic response (Beckmann et al., 2005; Damoiseaux et al., 2006; Smith et al., 2009). Magnetoencephalography (MEG) provides a powerful complement to fMRI because it captures the electrical properties of neural activity of the underlying networks (Barnes et al., 2016). The primary approach to identifying functional connectivity networks with MEG is to measure the synchronisation between the amplitude envelopes of oscillations within certain frequency bands, which yields networks of coordinated activity across spatially separate brain regions (de Pasquale et al., 2010; O'Neill et al., 2015; Sudre et al., 2017). This approach has characterised resting‐state networks (RSNs) that show a closely overlapping topography with those found using fMRI (Astle et al., 2015; Barnes et al., 2016; de Pasquale et al., 2010; Demuru et al., 2017). However, with both fMRI and MEG, the estimates of functional connectivity are typically derived by aggregating activity over large time windows, such as the full resting state acquisition period. This defines large‐scale networks as remaining stationary, whether during rest or task performance. Yet, recent work suggests large‐scale neural networks may synchronise their activity on a timescale of around 50–200 ms, including at rest, and switching far more quickly than can be captured using averages taken from time window methods (Baker et al., 2014; Colclough et al., 2017; Hindriks et al., 2016; Vidaurre et al., 2016; Vidaurre et al., 2017). This rapid synchronisation may help these large‐scale networks play an important role in supporting cognitive processes.

More recently, researchers have developed analytical techniques that fully capitalise on the high temporal resolution of MEG. An example is the use of Hidden Markov Modelling (HMM) to explore the temporal dynamics of these networks (Baker et al., 2014; Vidaurre et al., 2016; Vidaurre et al., 2017; Vidaurre et al., 2018). Instead of estimating the correlation between band‐limited amplitude envelopes separately in one large time window at a time, the HMM is a data‐driven method that identifies a sequence of “states”, whereby each state corresponds to a unique pattern of brain network activity and correlation that reoccurs at different points in time. For example, the pattern of amplitude envelope activity that characterises each state can be well‐estimated by pooling over the evidence provided by the many repeated visits to the state. This means that individual state visits can be potentially very short in time. Indeed, it has been shown that the HMM can identify network dynamics in resting MEG on ~100 ms time‐scale, which is much faster than can be investigated with traditional sliding time window approaches (Baker et al., 2014; Vidaurre et al., 2016). By quantifying the time‐series of MEG data as a sequence of transient states, the HMM provides information about the points in time at which each state is active, enabling the characterisation of the temporal dynamics of each state or network. Furthermore, the HMM allows the detection of rapid, transient organisation of brain networks and therefore represents a powerful tool for understanding how network dynamics can be influenced by, for example, genetic factors.

The application of these methods remains in its infancy. As a result, we have yet to establish how underlying neurobiological or molecular mechanisms can influence the formation of these large‐scale networks or their intrinsic temporal dynamics. Individuals with rare single‐gene mutations can represent a unique and interesting window to investigate the specific interactions between cellular and physiological processes involved in cognitive development. This is because, unlike neurodevelopmental disorders defined by behavioural impairments, they have a highly specific and known aetiology, common across all cases. Here, we studied a group of individuals with mutations in *ZDHHC9*, a rare recurrent cause of X‐linked intellectual disability (XLID; Han et al., 2017; Raymond et al., 2007; Schirwani, Wakeling, Smith, Study, & Balasubramanian, 2018; Masurel‐Paulet et al 2014). We previously found that individuals with *ZDHHC9* mutations are susceptible to a combined phenotype of speech and language impairments, cognitive difficulties and Rolandic Epilepsy (RE; Baker et al., 2015). We also found that individuals with *ZDHHC9* mutations have differences in structural connectivity which converge with the topography of *ZDHHC9* expression in the normal adult brain (Bathelt et al 2017). *ZDHHC9* encodes a palmitoylation enzyme involved in the posttranslational modification and intracellular trafficking of specific target substrates, including recruitment of receptors and ion channels to the synapse (Fukata & Fukata, 2010). Although multiple substrates may be relevant to neurodevelopmental disorders, one palmitoylation target is thought to be Post‐Synaptic Density protein 95 (PSD‐95) which is critical to activity‐dependent AMPA receptor availability (El‐Husseini et al., 2000a; El‐Husseini et al., 2000b; Bredt et al., 2010). Palmitoylation is itself activity‐dependent and influences synaptic stability across multiple timescales during development (Globa & Bamji, 2017; Kang et al., 2008; Kaur et al., 2016; Levy & Nicoll, 2017). Hence, the loss of *ZDHHC9* function and reduction in palmitoylation efficiency may alter dynamic aspects of postsynaptic activity, impacting on the emergence and stability of structural and functional networks supporting cognition. Because of this proposed physiological role of *ZDHHC9*, we predicted that mutations to the gene might result in a perturbation to the dynamic nature of large‐scale neural networks. Furthermore, we sought to assess whether those networks most altered would reflect the regional expression profile of the gene, whereby the temporal dynamics would be *altered maximally* in those networks in which the gene is highly expressed.

We investigated the dynamics of functional networks at rest and during an auditory oddball task in individuals with *ZDHHC9* mutations and control participants. We included both protocols because it is unclear whether the impact of a mutation upon neuronal dynamics would be most apparent when the system is at ‘rest’ or when under sensory stimulation. For our stimulation protocol, we chose an auditory oddball task because *ZDHHC9* participants have previously been shown to have impaired language development (Baker et al., 2015). Auditory oddball tasks, which require rapid habituation to a repeated standard stimulus and sensitivity to deviations from this stimulus, have been used to index auditory processing in developmental language disorders. These studies have established that language impairment is frequently associated with poorer auditory change detection and reduced sensitivity to phonetic and linguistic cues (Ahmmed, Clarke, & Adams, 2008; Baldeweg, Richardson, Watkins, Foale, & Gruzelier, 1999; Datta, Shafer, Morr, Kurtzberg, & Schwartz, 2010; Davids et al., 2011; Shafer, Yu, & Datta, 2011).

In sum, the aim of this study was to apply the novel HMM method to map dynamic neural abnormalities in individuals with *ZDHHC9* single‐gene loss‐of‐function mutations, during both rest and a passive auditory task, to determine whether these abnormalities relate to the expression profile of the gene. As we sought to assess whether regionally specific distribution of the *ZDHHC9* gene was associated with dynamic network differences in individuals with pathogenic variants of this gene, within our analysis we also compared neuronal dynamics with the expression profiles of other genes with known phenotypes that overlap with the *ZDHHC9* phenotype: *FMR1*, for which loss‐of‐function variants are the most common monogenic cause of intellectual disability (Bourgeois et al., 2009) and *FOXP2*, a gene linked with language disability (Vargha‐Khadem et al., 2005). To the best of our knowledge, this is the first study that has sought to establish whether there is a link between the dynamics of large‐scale brain networks, and the regional expression of a gene associated with synaptic regulation.

## 2 METHODS

### 2.1 Participants

Eight male participants with an inherited mutation to *ZDHHC9* (age in years: mean = 26.70, standard deviation (SD) = 13.74, range = 13.25–41.83) were compared to seven age‐matched male controls (age in years: mean = 27.23, SD = 14.05, range = 10.17–42.50; *t* = −0.74, *p* = .943). All control participants had no history of neurological disorders or cognitive impairments. For a detailed description of clinical and cognitive characteristics of the *ZDHHC9* group, compared to an age and IQ matched control group, refer to Baker et al. (2015). In brief, *ZDHHC9* participants had mild to moderate intellectual disability (standardised IQ scores: mean = 64.88, SD = 5.70, range = 57–73). Additionally, they displayed poor verbal fluency, difficulties with nonspeech oromotor control, and relatively strong receptive language abilities compared to expressive and written abilities. These communication characteristics distinguished the *ZDHHC9* group from an age‐ and IQ‐matched comparison group (Baker et al., 2015). Ethical approval for the study was granted by the Cambridge Central Research Ethics Committee (11/0330/EE).

### 2.2 MEG data acquisition and pre‐processing

**Figure 1:**
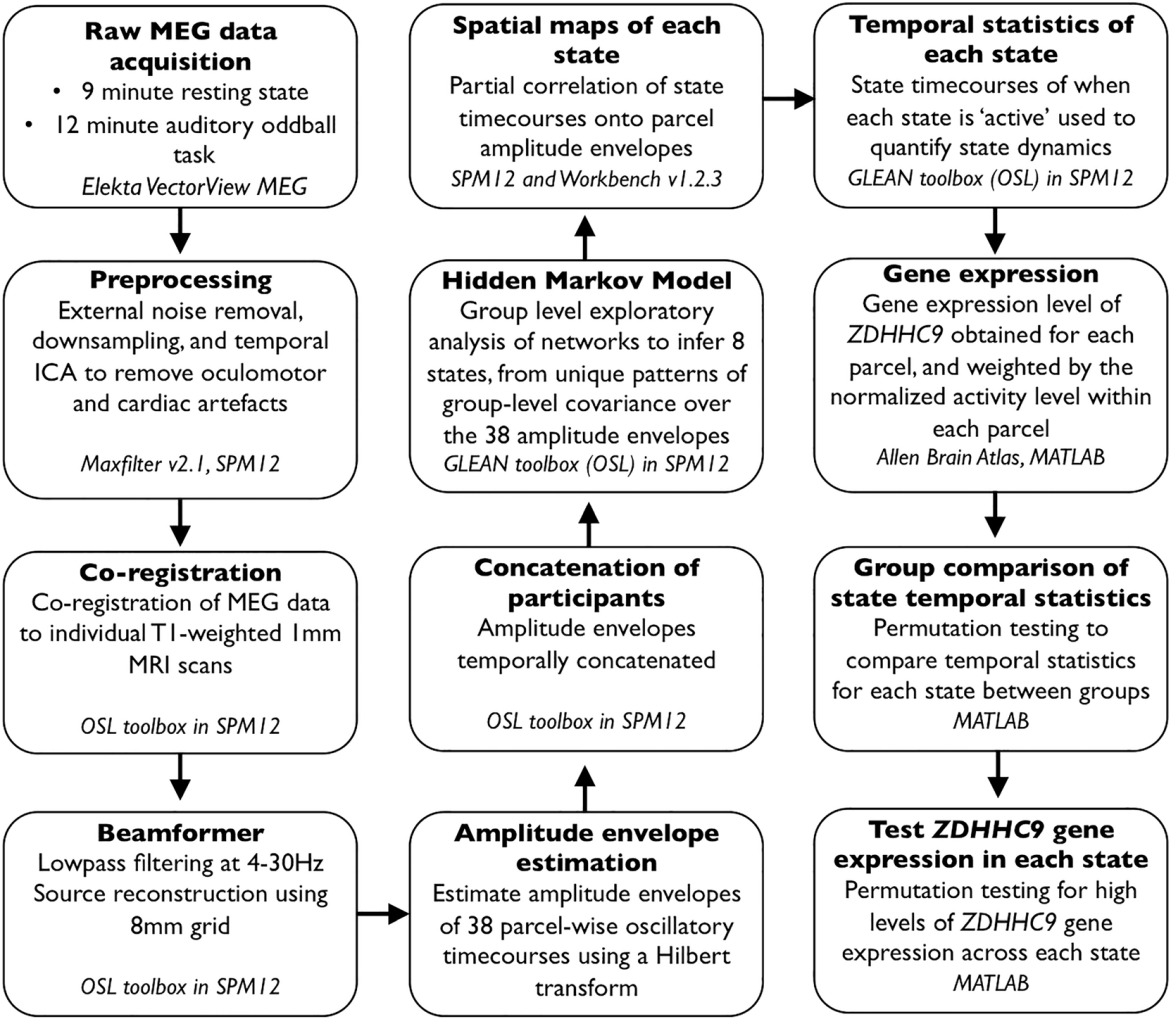
The processing and analysis pipeline used on the resting state and oddball task data

For each participant, we acquired data during 9 minutes of eyes‐closed resting state and a 12 minutes passive auditory oddball task. The data acquired from these two protocols were analysed separately, although using the same pipeline (Figure 1). During the resting‐state scan, participants were instructed to relax with their eyes closed and allow their mind to wander, without thinking of anything in particular, but without falling asleep. The oddball task was based upon a design by Cowan et al. (1993) using a roving standard stimulus and delivered in two 6 minutes blocks. Participants heard a sequence of tones at the same frequency, followed by a sequence of tones of a different frequency. The repeated tone in each sequence was, therefore, the standard stimulus, and first tone of the new sequence was the deviant tone. The frequency of the repeated standard stimuli altered randomly between three frequencies of 250, 500 and 1,000 Hz. The number of standard stimulus presentations that occurred in a single stimulus train varied randomly from 6 to 12. Tones were 50 ms in duration and the inter‐tone interval was 500 ms. Participants were asked to watch a silent nature documentary and to ignore the tones.

MEG data were acquired at the MRC Cognition and Brain Sciences Unit, Cambridge, UK. All scans were obtained using the 306‐channel high‐density whole‐head VectorView MEG system (Elekta Neuromag, Helsinki), consisting of 102 magnetometers and 204 orthogonal planar gradiometers, located in a light magnetically shielded room. Data were sampled at 1 kHz and signals slower than 0.01 Hz were not recorded. Before acquisition a 3D digitizer (Fastrack Polhemus Inc., Colchester, VA) was used to record the positions of five head position indicator (HPI) coils and 50–100 additional headshape points evenly distributed over the scalp, all relative to the participants’ fiducial points (nasion and left and right preauricular), which could later be co‐registered on MRI scans for source reconstruction. An ECG electrode was attached to each wrist to measure the pulse, and we attached bipolar electrodes to obtain horizontal and vertical electrooculograms (HEOGs and VEOGs). Head position was monitored throughout the recoding using the HPI coils.

External noise was removed from the MEG data using a signal‐space separation (sss) method, and adjustments in head position during the recording were compensated for using MaxMove software, both implemented in MaxFilter version 2.1 (Elekta Neuromag). Following this, data were converted to an SPM12 format and down‐sampled to 250 Hz. Continuous data were inspected and short sections with large signal jumps or artefacts were removed. A temporal independent component analysis (FastICA) was used in sensor space to remove artefacts arising from blinks, saccades and pulse‐related cardiac artefacts, by manual visual inspection. The criterion for removing artefacts was a high correlation between the topography of an independent component and one of the HEOG, VEOG and ECG channels. The artefacts were removed by subtraction. Approximately 2–4 components were removed per participant.

### 2.3 MEG source reconstruction, parcellation and envelope calculation

Each participant's continuous pre‐processed MEG data were co‐registered to their individual structural T1‐weighted MRI scan with a 1 mm image resolution, using the digitised headshape points and fiducials. For one *ZDHHC9* participant without an individual MRI scan, we used a T1‐weighted 1 mm template in MNI space. Co‐registration was run using the OSL toolbox in SPM12 (https://ohba-analysis.github.io/osl-docs/), which aligned the MRI coordinates with the fiducials and headshape points before positioning these data within the MEG sensors using the HPIs for source space analysis.

The MEG sensor data were then low pass filtered at 4–30 Hz, to focus on slower frequencies only. This is the frequency range at which amplitude correlations have been shown to produce robust resting‐state brain networks and better distinguish true from spurious connectivity (Colclough et al., 2016; Luckhoo et al., 2012). For each subject, source space activity was then estimated at every point of an 8 mm whole‐brain grid using a linearly constrained minimum variance (LCMV) scalar beamformer that combines information across both sensor types and accounts for the reduction in dimensionality induced by the signal‐space separation method (Van Veen et al., 1997; Woolrich, Hunt, Groves, & Barnes, 2011). The beamformer used a set of adaptive spatial filters to weight the sensor measurements into an estimate of neuronal activity using the cortical grid, by maximising the band‐passed signal at each grid point whilst minimising the signal passed from all other grid points. This process aimed to reduce the effect of signal leakage from neighbouring regions in order to provide a more accurate estimate of activity at each grid location. The beamformer repeated this process across all grid points to produce a whole‐brain reconstruction of source‐space activity.

Following the beamformer projection of the oscillatory signal into source space, the data across all grid points underwent parcellation into 38 regions of interest using an 8 mm mask, applying the method described in Colclough, Brookes, Smith, and Woolrich (2015) and Colclough et al. (2017). In brief, each parcel was identified using a symmetric orthogonalisation to produce parcel time‐courses which were as close as possible to the original time‐courses across voxels whilst minimising signal leakage, and correcting for source spread. The time‐course of oscillatory activity within each parcel was then obtained using a principal components analysis (PCA), in which the time‐courses from all voxels in the parcel were submitted to a PCA, where the first principal component explaining the majority of variance across voxels within each parcel was used as the time‐course estimate for that parcel using the spatial filter method (for full details of this method see Colclough et al., 2015).

The amplitude envelope of each parcel's oscillatory time‐course was then calculated using a Hilbert transform, which estimated the instantaneous signal amplitude at each time point. As in Baker et al. (2014), for computational efficiency the amplitude envelopes were downsampled to 40 Hz by temporally averaging within sliding windows with a width of 100 ms and 75% overlap between consecutive windows. The parcellation of the source‐space data acted to reduce the dimensionality of the oscillatory activity across all source locations into 38 time‐courses, to be submitted to the HMM. The amplitude envelopes were then concatenated temporally across all participants to produce a single dataset for the HMM analysis. This procedure assumed that the same set of microstates underpinned the HMM‐derived states across participants. The envelope data from each participant were demeaned and normalised by their variance prior to concatenation.

### 2.4 Group level exploratory analysis of networks (Hidden Markov Model)

We ran the HMM using the group‐level exploratory analysis of networks (GLEAN) toolbox (https://github.com/OHBA-analysis/GLEAN; Vidaurre et al., 2017). The HMM is specified a priori to derive a certain number of states from the data; we set the HMM to infer eight states, based on previous work suggesting that this number represents a reasonable trade‐off between providing a sufficiently rich description and avoiding an overly complex, and therefore hard to interpret, representation (Baker et al., 2014).

The HMM describes the time‐series of MEG amplitude envelope data as a temporal sequence of states, where each state describes the data as coming from a unique 38‐dimensional multivariate normal distribution, defined by a covariance matrix and a mean vector. Each state, therefore, corresponds to a unique pattern of amplitude envelope activity and covariance that reoccurs at different points in time; where the HMM state time‐courses define the points in time at which each state was “active”. The estimated state time‐courses, corresponding to a binary sequence showing the points in time when that state was most probable, were obtained using the Viterbi algorithm (Rezek & Roberts, 2005). To produce spatial maps of the changes in amplitude envelope activity associated with each state, the state time‐courses were partially correlated onto whole‐brain parcel‐wise amplitude envelopes concatenated across subjects. The resulting state maps show the brain areas whose amplitude envelopes increase or decrease when the brain visits that state, compared to what happens on average over time.

From the state time‐courses, we were able to quantify the temporal characteristics of each state in terms of four measures of interest: (a) Fractional occupancy: The proportion of time each state was active, (b) Number of occurrences: The number of times a state was active, (c) Mean life time: The average time spent in a state before transitioning to another state, and (d) Mean interval length: the average duration between recurring visits to that state. Because these temporal properties reflect the duration and frequency of the coordinated oscillatory activity characterising each state, it was of particular interest to examine whether the expression of the *ZDHHC9* mutation affected the dynamics of these connectivity patterns. To test this hypothesis, we compared each of these temporal characteristics between groups using non‐parametric permutation testing. For each temporal property, on each of the eight states, we randomly allocated participants’ values to two groups and calculated the mean difference between these groups. This process was repeated 10,000 times to generate a null distribution of the mean difference between groups that would be expected when group membership was random. The actual group difference in state dynamics was then compared to the permuted null distribution to assess whether this group difference was greater than what we would have expected by chance, and the position of the observed mean difference in the null distribution provided the *p*‐value. All *p*‐values were corrected for multiple comparisons using the Benjamini–Hochberg procedure for false discovery rate (FDR) correction, which is appropriate for maximising power with a large number of tests (Hochberg & Benjamini, 1990).

### 2.5 Testing the association between gene expression and state dynamics

Gene expression data were obtained from the Allen Brain Atlas Human Brain public database (http://human.brain-map.org; Hawrylycz et al., 2012). These datasets were based on microarray analysis of postmortem tissue samples from six human donors aged between 18 and 68 years with no known history of neuropsychiatric or neurological conditions. MRIs and transformations from individual donor MR space to MNI coordinates were also obtained from the Allen Brain Atlas website. Gene expression (RNA) values were averaged across these six donors and mapped onto areas of the Desikan–Killiany (DK) parcellation of the MNI brain. This provided 68 cortical regional *ZDHHC9* expression values (34 in each hemisphere). We repeated the same procedure for two other genes, both with well‐documented overlapping phenotypes with our group. The first was *FMR1*. Loss of function variants of this gene are the most common monogenic cause of intellectual disability (Bourgeois et al., 2009), and thus overlaps with the formal diagnosis of our participants. The second, *FOXP2*, is gene linked with language disability (Vargha‐Khadem et al., 2005), and thus overlaps with the oromotor language symptoms of our group.

Next, we tested whether the impact of the mutation on dynamic transient networks was predicted by the expression profile of these genes. Specifically, does regional variation in the expression of a gene predict which networks ought to be most disrupted by the mutation? Simply performing a spatial correlation between the gene expression and the activity pattern across parcels would not be viable because the networks are highly variable. Instead, we calculated a gene expression value for each network, by taking the 20 most active parcels for each network and summing the level of gene expression. The 20 most active parcels of each network were defined as those with the strongest contribution to that network, which was indexed by the absolute partial correlation of the state‐time courses with the amplitude envelopes in each parcel. Twenty parcels were selected by exploratory analysis as this best reflected an optimal trade‐off between peak activation and spatial state distribution.

The sum of these values thus provided an expression value for the *ZDHHC9*, *FMR1* and *FOXP2* gene for each network. A null distribution was generated from the random selection of 20 parcel expression values across all 68 cortical parcels. This was done for 10,000 iterations to form the null distribution expression values across each state. By comparing the gene expression value for each network (found from the 20 most active parcels) to this null distribution, we were able to identify the level of gene expression within each network, relative to what would be expected by chance. Once we had these values for each state and each gene, we could test whether the degree of expression of a particular gene would predict the magnitude of the dynamic neural measures using Spearman's correlations.

## 3 RESULTS

### 3.1 Dynamic transient networks derived from the HMM in the resting state data

We first ran the HMM on resting data from the control and *ZDHHC9* participants separately, to verify that similar networks were present in each group by visual inspection. The spatial patterns of activity in each state strongly overlapped between groups, and were similar to established resting‐state networks at slower time‐scales (e.g., Damoiseaux et al., 2006; Smith et al., 2012) and to Baker et al. (2014).

**Figure 2:**
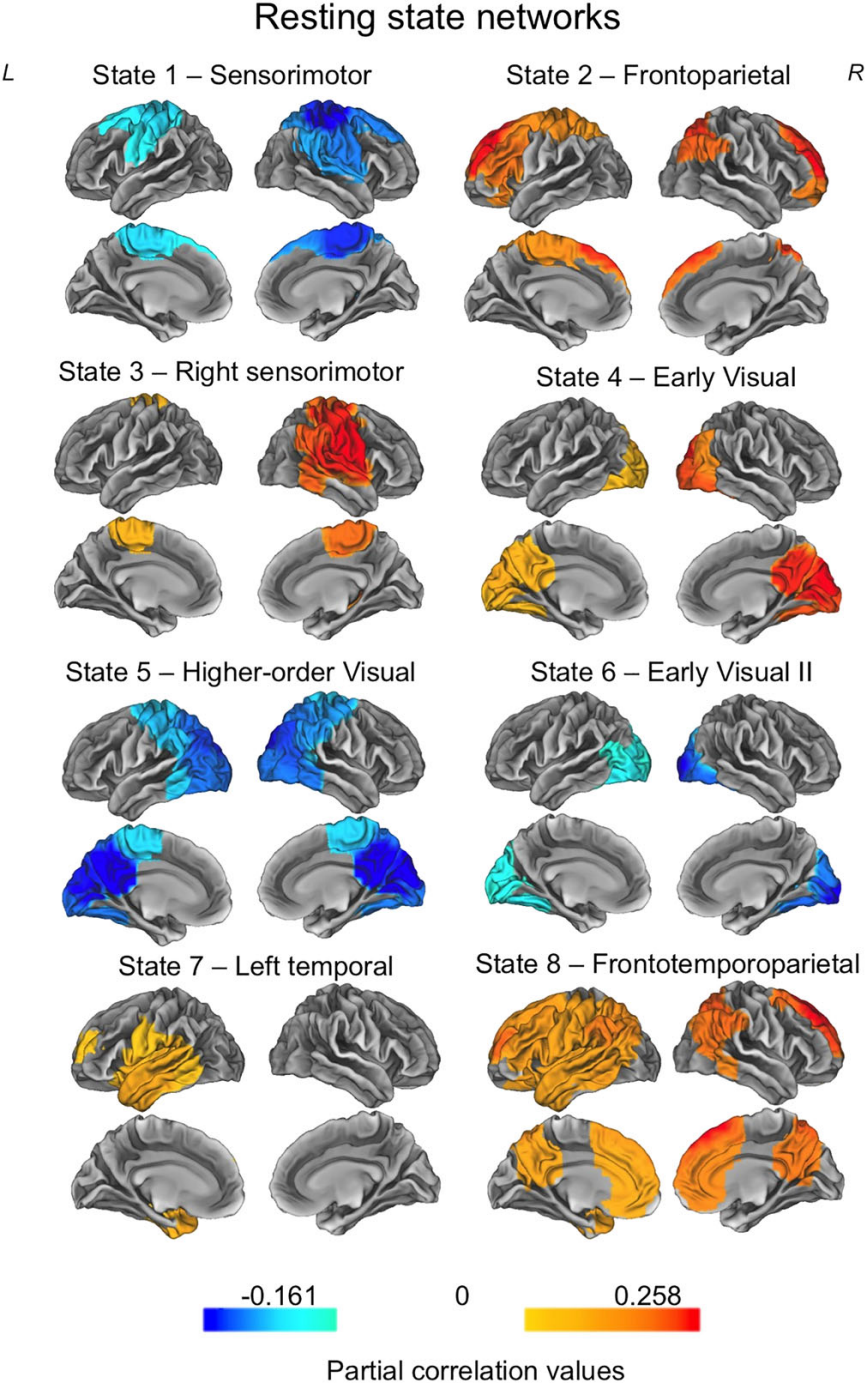
The eight states inferred from the resting state data. Each map shows the partial correlation between each state time course and the parcel‐wise amplitude envelopes. The partial correlation values have been thresholded to show correlation values above 70–80% of the maximum correlation for each state, and the colour maps are normalised relative to all states [Color figure can be viewed at http://wileyonlinelibrary.com]

After confirming that we obtained similar spatial patterns of activity in both groups, we ran the HMM on all participants’ concatenated data to derive common states across both groups, on which we could then compare the groups on temporal measures of interest. Table 1 and Figure 2 shows the spatial maps of each network derived from the resting state data. In the spatial maps, the red/yellow colours represent brain areas in which the amplitude envelope increases when the brain visits that state and blue colours represent brain areas in which the amplitude envelope decreases in that state. The states included sensorimotor networks (State 1 and State 3), a frontoparietal network (State 2), early visual networks (State 4 and State 6), a higher‐order visual network (State 5), a left temporal network (State 7) and a distributed frontotemporoparietal network (State 8).

**Table 1:** Descriptive statistics of the temporal properties of each network, across the control and *ZDHHC9* participants

	Control								ZDHHC9							
	Fractional occupancy		Number of occurrences		Mean lifetime		Mean interval length		Fractional occupancy		Number of occurrences		Mean lifetime		Mean interval length	
	Mean	SD	Mean	SD	Mean	SD	Mean	SD	Mean	SD	Mean	SD	Mean	SD	Mean	SD
*Resting state*																
State 1—Sensorimotor	19.17	5.29	420.14	103.48	182.61	27.22	0.90	0.38	18.18	8.19	333.88	124.96	210.29	63.41	1.20	0.57
State 2—Frontoparietal	0.33	0.30	7.71	8.32	223.87	267.67	95.30	89.49	0.40	0.50	5.75	6.25	110.54	121.94	147.16	208.53
State 3—Right sensorimotor	8.46	2.79	196.57	48.04	156.72	23.71	1.91	0.59	11.40	5.63	245.38	95.14	160.27	29.76	1.54	0.56
State 4—Early visual	10.01	2.92	243.00	59.99	153.94	36.95	1.59	0.49	16.93	5.72	320.38	104.11	210.47	55.39	1.20	0.41
State 5—Higher‐order visual	18.94	11.24	275.86	93.12	287.90	141.73	1.53	0.59	14.80	9.15	198.38	124.28	307.93	130.13	5.80	8.20
State 6—Early visual II	29.17	12.62	563.57	145.77	208.72	51.95	0.59	0.24	20.43	9.07	401.50	130.53	191.89	49.21	0.88	0.33
State 7—Left temporal	8.68	2.92	220.14	54.82	139.90	27.31	1.73	0.49	11.41	5.76	250.25	112.14	159.96	51.90	1.55	0.59
State 8—Frontotemporoparietal	5.22	3.24	97.43	44.19	202.97	56.16	4.74	1.78	6.43	4.66	105.38	55.27	203.18	93.75	6.72	9.19
*Oddball task*																
State 1—Parietal	4.31	9.05	37.43	73.57	105.78	146.26	128.96	68.34	12.07	12.61	68.19	74.73	315.48	259.69	57.68	56.94
State 2—Frontoparietal	2.62	2.60	11.64	8.39	222.03	73.57	38.96	37.60	13.40	19.29	20.61	10.71	731.25	1021.40	20.26	12.31
State 3—Fronto‐occipital	10.17	7.60	89.36	66.26	224.41	75.67	12.91	16.74	11.11	6.94	100.53	69.57	319.86	44.20	26.03	26.96
State 4—Frontotemporal	21.32	22.01	69.21	33.46	398.80	250.83	12.91	4.97	15.95	12.91	51.86	17.47	352.22	132.43	10.22	10.06
State 5—Right temporoparietal	13.28	15.58	94.50	113.89	198.52	166.69	70.38	67.02	17.72	12.63	138.53	116.29	264.83	143.91	40.27	50.34
State 6—Bilateral temporal	18.42	8.47	99.57	27.55	323.73	78.18	2.45	1.07	8.33	5.12	61.47	28.13	324.60	102.58	19.26	24.25
State 7—Frontoparietal II	11.70	10.98	82.07	63.75	255.65	139.93	25.71	38.25	13.03	9.02	90.69	56.92	291.51	149.83	25.82	26.59
State 8—Fronto‐occipital II	19.05	10.49	99.29	31.10	301.25	107.02	2.97	2.00	8.40	6.26	56.19	26.83	309.62	101.75	19.83	24.67

We next characterised the temporal properties of each state in terms of its fractional occupancy, number of occurrences, mean life time and mean interval length. We tested whether these temporal properties differed between the *ZDHHC9* and control group using nonparametric permutation testing, as described in section 2.4, and corrected for multiple comparisons using the Benjamini–Hochberg false discovery rate procedure. On the fractional occupancy measure only an early visual network (State 4) distinguished the two groups, whereby the *ZDHHC9* group spent a higher percentage of time in this state than the control group (actual group mean difference 6.92%; *p* = .01). No other states differed significantly between the groups in fractional occupancy. In terms of the number of occurrences, none of the eight states differed between the groups. The mean lifetime of the states differed between the groups on the early visual network (State 4), where the duration spent in this state was longer for the *ZDHHC9* participants than controls, but this was nonsignificant following multiple comparison correction. No other states differentiated the groups. Table 1 presents the descriptive statistics across all states, and Table 2 summarises the group differences and statistical results.

**Table 2:** The results of the statistical tests for between‐group differences in temporal dynamics across the resting state and oddball tasks

	Group differences (control‐ ZDHHC9)							
	Fractional occupancy		Number of occurrences		Mean lifetime		Mean interval length	
	Mean	*p*	Mean	*p*	Mean	*p*	Mean	*p*
*Resting state*								
State 1—Sensorimotor	0.99	.798	86.27	.170	−27.68	.312	−0.30	.255
State 2—Frontoparietal	−0.07	.758	1.96	.592	113.33	.334	−51.85	.531
State 3—Right sensorimotor	−2.94	.254	−48.80	.268	−3.54	.815	0.36	.253
State 4—Early visual	−6.92	.010*	−77.38	.098	−56.53	.031	0.39	.124
State 5—Higher‐order visual	4.14	.428	77.48	.197	−20.03	.754	−4.27	.213
State 6—Early visual II	8.74	.145	162.07	.041	16.83	.520	−0.29	.060
State 7—Left temporal	−2.73	.294	−30.11	.533	−20.07	.403	0.18	.522
State 8—Frontotemporoparietal	−1.21	.599	−7.95	.765	−0.21	.999	−1.99	.976
*Oddball task*								
State 1—Parietal	−7.76	.261	−30.77	.482	−209.70	.089	71.30	.080
State 2—Frontoparietal	−10.78	.139	−8.97	.118	−509.22	.067	18.70	.361
State 3—Fronto‐occipital	−0.94	.805	−11.17	.760	−95.44	.020	−13.10	.259
State 4—Frontotemporal	5.38	.624	17.35	.274	46.58	.695	−4.11	.434
State 5—Right temporoparietal	−4.44	.577	−44.03	.507	−66.31	.459	30.10	.391
State 6—Bilateral temporal	10.09	.023	38.10	.032	−0.87	.988	−16.80	.018
State 7—Frontoparietal II	−1.32	.814	−8.62	.794	−35.86	.660	−0.11	.901
State 8—Fronto‐occipital II	10.64	.048	43.09	.021	−8.38	.882	−16.90	.042

*Note*: Statistical significance was derived using permutation testing and corrected for multiple comparisons as described in the Methods. *Significance following multiple comparison correction at the *p* < .05 level and **Significance at the *p* < .001 level.

### 3.2 Dynamic transient networks derived from the HMM in the oddball data

**Figure 3:**
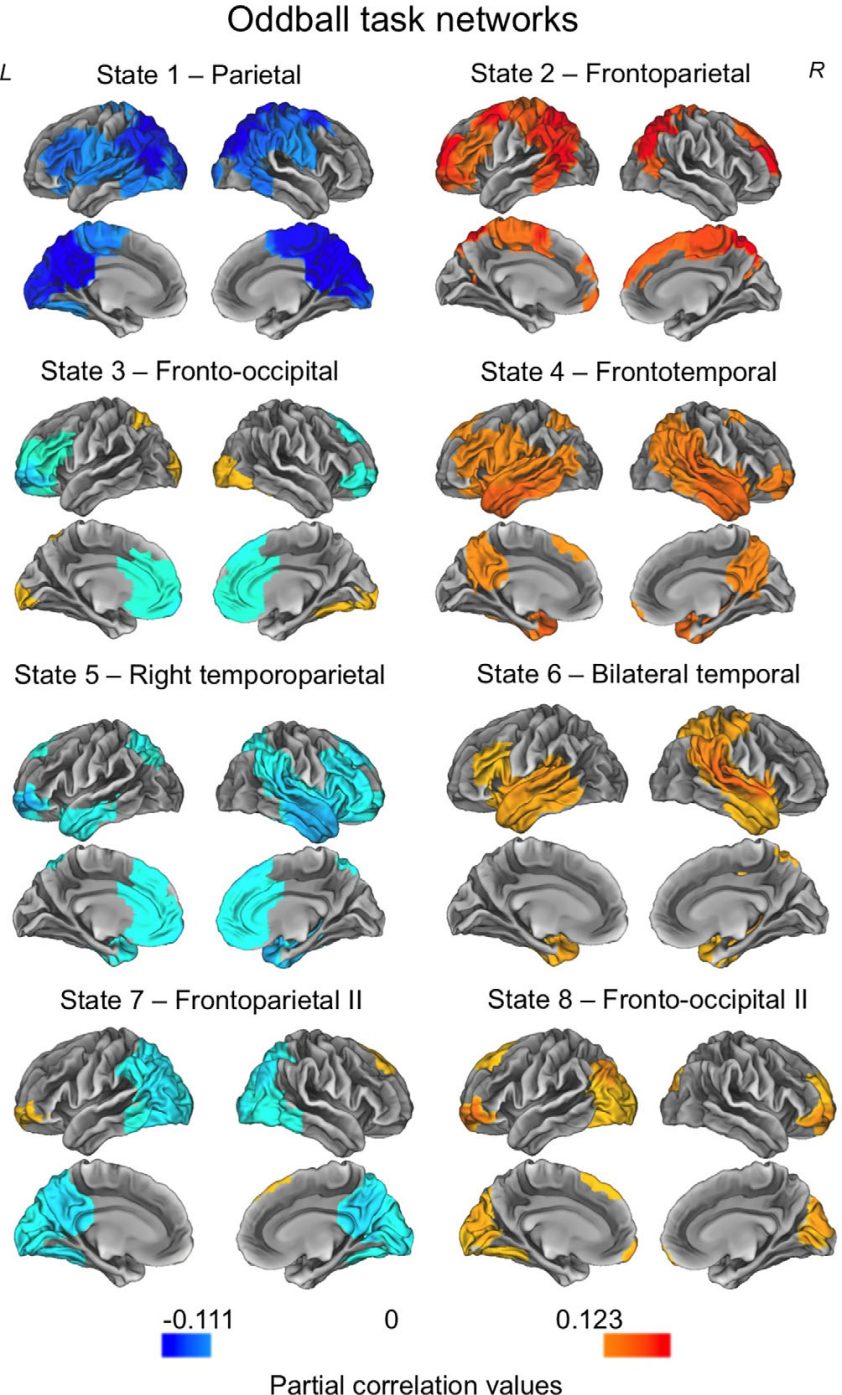
The eight states inferred from the oddball task data. Each map shows the partial correlation between each state time course and the parcel‐wise amplitude envelopes. The partial correlation values have been thresholded to show correlations above 60–80% of the maximum correlation for each state. The colour maps are normalised relative to all states [Color figure can be viewed at http://wileyonlinelibrary.com]

We applied the same analysis pipeline as described in the Methods to the oddball dataset. Two *ZDHHC9* participants were excluded from the oddball analysis due to failed source reconstruction, but this did not affect the age‐matching of the samples (*ZDHHC9* group age in years: mean = 27.11, SD = 12.80, range = 13.25–41.83; comparison with control group, *t* = −0.015, *p* = .988). As with the resting state data, we first established whether similar networks were generated by conducting the analysis on each group separately. Following this, we combined the two groups into a single analysis. The eight states derived from the oddball dataset can be seen in Figure 3. The states included a parietal network (State 1), frontoparietal networks (State 2 and State 7), frontooccipital networks (State 3 and State 8), a frontotemporal network (State 4), right temporoparietal network (State 5) and bilateral temporal network (State 6).

We next examined whether the temporal properties of the networks distinguished the *ZDHHC9* and control groups. As with the resting state data, we tested this using nonparametric permutation testing, as described in the Methods, and corrected for multiple comparisons using the Benjamini–Hochberg false discovery rate procedure. Table 1 summarises the descriptive statistics and Table 2 presents the statistical comparisons between groups. In short, the groups consistently differed in the bilateral temporal network (State 6) and frontooccipital network (State 8) in fractional occupancy, number of occurrences and mean interval length. The *ZDHHC9* group spent a lower proportion of time in both states, with a lower number of occurrences and shorter mean interval length. However, these group differences were not significant after correcting for multiple comparisons.

In summary, we were able to derive networks from both the resting‐state and oddball data, which showed good similarity to known functional connectivity networks obtained from fMRI, and time‐averaged MEG data. The two groups differed significantly in an early visual network in the resting state data, with *ZDHHC9* participants spending a longer period of time in this state. In the oddball task data, bilateral temporal and frontooccipital networks showed descriptive between‐group differences, with lower activation indices in the *ZDHHC9* group, but these differences were not significant following multiple comparison correction. The next step in our analysis was to explore the level of *ZDHHC9*, *FMR1 and FOXP2* gene expression in the HMM‐derived networks, and test whether relative between‐group differences in network dynamics were associated with the relative expression profile of the gene.

### 3.3 Statistical association between gene expression and dynamic network group differences

Finally, we sought to determine whether *ZDHHC9*, *FMR1 and FOXP2* gene expression would predict group differences in neuronal dynamics across networks. Using gene expression data from the Allen Atlas (Hawrylycz et al., 2012), we identified the level of *ZDHHC9*, *FMR1* and *FOXP2* gene expression within each of the 68 cortical parcels. For each network, we could then calculate a gene expression value across the top 20 most active parcels in that state. We then used a null distribution, derived using a permutation procedure to identify the likelihood of this expression value, relative to chance. This approach is described in the Methods section.

We used a Spearman's rank‐order correlation to test for a statistical association between the magnitude of the gene expression effect, indexed by the *p*‐value of gene expression in each state from our permuted distributions, and the magnitude of the group difference, indexed by the *p*‐value of the group difference on each temporal measure (from the group‐level permutation testing). Because the *p*‐values were derived from permuted distributions they provided a measure of the *size* of the gene expression effect and group difference in each state, allowing us to test for a relationship between the extent to which network dynamics distinguished the groups and the level of gene expression. Overall, this analysis approach, therefore, allowed us to first identify the level of gene expression in each state, and to then test the association between these gene expression levels and the magnitude of group differences in neuronal dynamics across networks.

The resting‐state data showed a significant association between the magnitude of dynamic neural differences and *ZDHHC9* gene expression. The group difference in fractional occupancy, number of occurrences, and mean interval length was strongly associated with *ZDHHC9* gene expression across states (fractional occupancy: *r*_s_ = .738, *p* = .046; number of occurrences: *r*_s_ = .857, *p* = .012; mean interval length: *r*_s_ = .833, *p* = .0154). This demonstrated that these dynamic properties of resting state networks were significantly associated with the strength of *ZDHHC9* expression, whereby higher gene expression was associated with a larger impact of the mutation on the dynamic properties across networks. Mean lifetime was not significantly associated with *ZDHHC9* gene expression (mean lifetime: *r*_s_ = .452, *p* = .267).

We further tested whether these same relationships were present with two other genes, *FMR1* and *FOXP2*, associated with overlapping phenotypes. There was no significant relationship between the profile of *FMR1* expression and any of the neural measures (fractional occupancy: *r*_s_ = .262, *p* = .536; number occurrences: *r*_s_ = 0, *p* = 1; mean lifetime: *r*_s_ = .262, *p* = .536; mean interval length: *r*_s_ = 0, *p* = 1). The group difference in number of occurrences was significantly associated with *FOXP2* gene expression (*r*_s_ = .762, *p* = .037) but not with fractional occupancy, mean lifetime or mean interval length (fractional occupancy: *r*_s_ = .119, *p* = .793; mean lifetime: *r*_s_ = −.0238, *p* = .977; mean interval length: *r*_s_ = .691, *p* = .0694). This provides some evidence that another gene associated with a language disorder is also related to these dynamic measures (*FOXP2*), but not another gene identified as a common monogenic cause of intellectual disability (*FMR1*).

**Figure 4:**
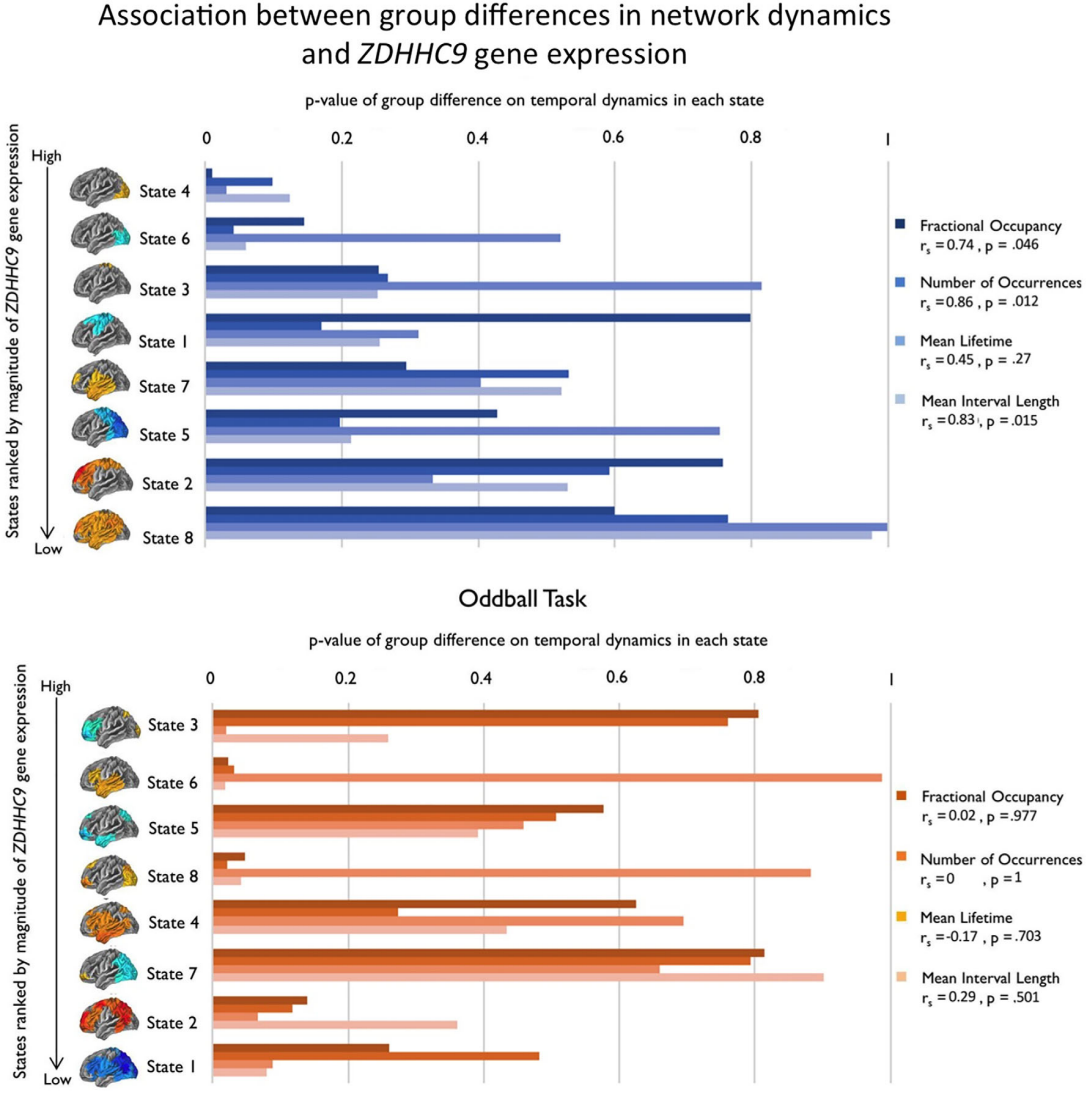
The association between the magnitude of the group difference in network dynamics and the level of *ZDHHC9* gene expression, across all temporal measures and states. The states are ranked by the level of gene expression on the *y*‐axis, in which states with smaller *p*‐values derived from the permutation testing have higher levels of gene expression. The *x*‐axis shows the *p*‐value of the group difference in network dynamics on each temporal measure, whereby smaller *p*‐values denote larger group differences. The plot for the resting state data demonstrates that the extent to which the dynamic properties differed between groups was associated with a larger magnitude of gene expression across all states. There was no significant across‐state association between gene expression and dynamic network group differences in the oddball task [Color figure can be viewed at http://wileyonlinelibrary.com]

In the oddball data, none of the group differences was significantly related to *ZDHHC9* or *FMR1* expression (*ZDHHC9*: fractional occupancy: *r*_s_ = .024, *p* = .977; number of occurrences: *r*_s_ = 0, *p* = 1; mean lifetime: *r*_s_ = −.167, *p* = .703; mean interval length: *r*_s_ = .286, *p* = .501; *FMR1*: fractional occupancy: *r*_s_ = .595, *p* = .132; number of occurrences: *r*_s_ = .405, *p* = .327; mean lifetime: *r*_s_ = 0, *p* = 1; mean interval length: *r*_s_ = .309, *p* = .462). The group difference in mean lifetime was significantly associated with *FOXP2* gene expression (mean life time: *r*_s_ = −.738, *p* = .046) but not with fractional occupancy, number of occurrences or mean interval length (fractional occupancy: *r*_s_ = −.143, *p* = .752; number of occurrences: *r*_s_ = −.024, *p* = .977; mean interval length: *r*_s_ = −.240, *p* = .582). Figure 4 presents the *ZDHHC9* gene expression results across the resting state and oddball task data.

In sum, we tested the association between group differences in dynamic network properties and gene expression across the resting state and oddball task networks. At rest the size of the group difference in fractional occupancy, number of occurrences, and mean interval length was systematically associated with the level of *ZDHHC9* gene expression. There were also some associations between these metrics and *FOXP2* expression. However, there were no links with *FMR1* expression.

## 4 DISCUSSION

Little is known about variability in dynamic network properties or the underlying physiological mechanisms that drive this variability. In the current study, we sought to characterise the dynamics of functional connectivity networks in individuals with the *ZDHHC9* gene mutation, a single‐gene developmental disorder associated with a homogenous phenotype of intellectual, language, and attentional impairments. We examined network dynamics using a Hidden Markov Model (HMM), a data‐driven method that captures functional networks on a millisecond timescale and quantifies their dynamics. At rest, an early visual network was active for a significantly longer proportion of time in individuals with the *ZDHHC9* mutation than in age‐matched controls. Importantly, across all resting‐state networks the size of the mutation effect was strongly associated with the expression profile of the gene: the greater the gene expression within a particular network, the stronger the impact of the mutation on the dynamics of that network. This was not true for another gene commonly associated with intellectual disability, *FMR1*. However, there were weaker associations with the expression profile of *FOXP2*, a gene associated with language impairment (Vargha‐Khadem et al., 2005). During the auditory oddball task, there were no consistent group effects that survived multiple comparisons correction, and there was no consistent correspondence between network dynamics and gene expression. In summary, in resting‐state MEG data we identified the impact of the gene mutation on the dynamics of specific networks, and crucially, the graded impact of this mutation was predicted by the level of gene expression across networks. However, these gene effects were not reliably detected in task‐positive data.

At rest, the higher proportion of time spent in the early visual network in the *ZDHHC9* group suggested less dynamic regulation of transitioning into and out of this brain state than in control participants. One interpretation of this finding is that slower engagement of certain functional networks at rest may present a constraint on higher‐level cognitive abilities (Basten, Hilger, & Fiebach, 2015). A growing number of studies have argued that the rapid and transient organisation of multiple network configurations at rest necessarily provides the flexibility to adapt to the changing demands of cognitive processing, by providing a continuous “dynamic repertoire” of states to quickly engage the optimal network configuration for a given task (e.g., Bressler & Tognoli, 2006; Deco, Jirsa, & McIntosh, 2011). Aligning with this, recent work by Schultz and Cole (2016) observed that smaller changes in functional connectivity patterns between rest and distinct tasks correlated with higher levels of fluid intelligence. Given the intellectual difficulties in individuals with the *ZDHHC9* gene mutation (Baker et al., 2015), it is plausible that slower dynamic transitions between resting state networks may constrain the emergence of higher level cognitive abilities through reduced efficiency in coordinating relevant network configurations (e.g., Hearne, Mattingley, & Cocchi, 2016; Song et al., 2008; van den Heuvel, Stam, Kahn, & Hulshoff Pol, 2009).

However, it is notable that the *ZDHHC9* and control group were only distinguishable on the resting‐state dynamics of this early visual network, rather than more distributed frontoparietal or temporal networks which we may have expected to mediate the intellectual and language difficulties in the disorder (e.g., Langeslag et al., 2013). Although functional variations in the occipital cortex have been linked to variability in intelligence (Jung & Haier, 2007) there is weak meta‐analytic support for this view (Basten et al., 2015). A plausible interpretation for the present study is that these sensory spatiotemporal patterns of activity were more stable across participants (Lee & Frangou, 2017; Moussa et al., 2012), allowing more reliable measurement of between‐group differences in temporal dynamics. The relationship between visual network connectivity and performance across varying language, working memory and reasoning tasks (Schultz & Cole, 2016) suggests that early visual network activation may reflect nonspecific engagement in the attentional demands of the study.

Increased alpha power in the early visual state in the *ZDHHC9* group could have also contributed to this group difference, which is a possibility we cannot rule out in the present study. However, we do not think this drives the observed group difference. First, we may have expected gross disparities in alpha power to cause more ubiquitous effects across states, but the observed group difference was specific: A higher proportion of time spent in the early visual state in *ZDHHC9* than control participants. Second, our methods normalised within participants to derive the dynamic measures, meaning overall basic differences between the groups (such as in amplitude differences driven by alpha power) were controlled for in the group comparisons. Third, it is of note that *ZDHHC9* expression is higher in primary visual cortex than in other neocortical areas during two important developmental phases: fetal mid‐gestation and adolescence (Kang et al., 2011; http://hbatlas.org). This provides some evidence for spatiotemporal specificity of *ZDHHC9* loss of function impacting on neurophysiological function and the development of visual systems. As such, whilst we cannot rule out potential confounders in the observation of this effect in the early visual state, the slower dynamics may nonetheless be informative about the properties of resting‐state networks in this disorder.

An important advance in the current study was our demonstration that the integrity of network dynamics at rest strongly overlap with regional differences in *ZDHHC9* gene expression, and to a lesser extent *FOXP2* expression. We predicted that state dynamics would be most altered in regions of elevated gene expression, with these regionally specific abnormalities potentially arising due to reduced palmitoylation and postsynaptic dysfunction (Fukata & Fukata, 2010; El‐Husseini et al., 2000a, 2000b). Our resting state results strongly aligned with this expectation: At rest the magnitude of group differences in dynamic network properties were associated with the level of *ZDHHC9* gene expression across networks, suggesting that higher expression of the *ZDHHC9* mutation has a relatively direct effect on neuronal dynamics. The significantly elevated levels of *ZDHHC9* gene expression in resting‐state networks showing case–control differences, suggest that reduced capacity for activity‐dependent postsynaptic change, resulting from reduced palmitoylation, may be a contributory mechanism to slower network dynamics (El‐Husseini et al., 2000a, 2000b).

In addition to testing the link between *ZDHHC9* gene expression and the magnitude of between‐group dynamic differences across networks, we sought to understand the specificity of this link by testing this association with two further genes associated with phenotypes overlapping with that of *ZDHHC9*: *FMR1* and *FOXP2*. The partial overlap between the magnitude of group differences in network dynamics and the expression profile of *FOXP2*, but not *FMR1*, suggests that this gene‐brain relationship reflects disruption to a molecular pathway relevant to the language impairment profile that our participants demonstrate (Baker et al., 2015). To extend these observations, future studies could investigate network dynamics in individuals with variants in *FOXP2*, and variants in other genes with clearly defined synaptic functions and converging phenotypes (e.g., *GRIN2A*). Parallel investigations in non‐human experimental models are needed to establish a fully mechanistic account. Importantly, the current study therefore provides only proof‐of‐principle of this methodological approach and the relationship between gene expression and neuronal dynamics. Future studies with more heterogeneous groups and significantly larger sample sizes are necessary to assess how different genes are differentially related to network dynamics, and the pathway between these gene‐brain relationships and variation in phenotype.

There was a striking difference between the resting and oddball data, in that there was no association between *ZDHHC9* gene expression and network dynamics in the oddball task. One possibility is that mutation effects do affect task network dynamics, but that the current sample had insufficient power to detect them. For example, we observed differences in the bilateral temporal and frontooccipital network dynamics between the control and *ZDHHC9* group. Whilst these effects were not statistically significant, they align with findings in other populations with partially overlapping phenotypes of language impairment who have also shown reduced sensitivity to linguistic and nonlinguistic auditory contrasts (Davids et al., 2011), which are in turn predictive of subsequent language development (Port et al., 2016). Furthermore, weaker functional integration in static language‐relevant connectivity networks is also a cardinal feature of children with Rolandic epilepsy and comorbid language delays, features observed in the *ZDHHC9* sample (Besseling et al., 2013; McGinnity et al., 2017). The reason why these effects were not robust enough to survive our multiple comparisons correction might be linked to the small number of cases within our sample. However, this would not explain the complete lack of relationship with gene expression in the oddball data, suggesting that this is not simply a power issue. A second alternative is that the measures of neuronal dynamics used here are not the most sensitive for task‐positive datasets. There may be dynamic network properties, such as the temporal overlap of activation between states, which we did not detect here but are nevertheless susceptible to disruption from postsynaptic dysfunction. In essence, different neuronal mechanisms could reflect the behavioural phenotype that we observe, but these are not directly influenced by the known aetiology of the *ZDHHC9* mutation. Importantly, these questions also point to the need for future work to test the correspondence between the dynamic measures used here and standard “stationary” network analyses and electrophysiological measures, to assess how dynamic measures extend our understanding of behavioural phenotypes.

There are important broader limitations to the current study. First, the sample size of studies of single‐gene mutations is inherently limited due to the rarity of these disorders. The present study included all known UK families diagnosed with *ZDHHC9*‐associated XLID at the time of recruitment, but the small sample size necessitates replication of these results in a larger sample. However, similar studies of small groups with a common aetiology have generated important hypotheses about neural and genetic pathways to a disorder, such as Fragile X syndrome (Diersson & Ramakers, 2006; van der Molen, Stam, & van der Molen, 2014). The present study further adds dynamic network irregularities as neurobiology‐relevant metrics associated with cognitive and/or language impairment.

Second, the control group was age‐matched to the *ZDHHC9* group but had age‐typical IQ and language abilities. It is therefore unclear whether the observed dynamic network differences are specifically tied to the combined phenotype of low IQ and language impairment, the aetiology of *ZDHHC9* mutations, or reflect more general correlates of low cognitive ability. The partial overlap between network dynamics and *FOXP2* expression suggests that the dynamic network differences may reflect disruptions to molecular pathways relevant to language impairment phenotypes, but not *specific* to the *ZDHHC9* gene. Rather, different genes may differentially affect network dynamics through their impact on neuronal excitability, but the current study cannot distill the specificity of these effects to the *ZDHHC9* mutation. It is important for future studies to explore dynamic functional connectivity in individuals with different aetiologies but matched on partially or fully overlapping phenotypes, to draw firmer conclusions about the relationship between causal pathways, dynamic network properties and behavioural phenotypes. In particular, relating dynamic network properties to individual differences in cognition during development may assess whether dynamic network irregularities present a risk factor for specific profiles of cognitive deficits.

Whilst there is a strong tradition of examining the impact of genetic effects on static functional connectivity networks through either heritability (Colclough et al., 2017; Demuru et al., 2017; Fu et al., 2015; Posthuma et al., 2005) or associations between gene expression and amplitude envelope coordination in resting‐state activity (Gordon, Devaney, Bean, & Vaidya, 2015; Jamadar et al., 2013; Wang et al., 2015), there remains a scarcity of work examining genetic effects on the fast transient dynamics of functional connectivity networks. Investigating network dynamics is particularly compelling to understand how gene expression perturbs communication within large scale brain networks, and downstream impacts on cognitive abilities, due to the profound impact of genetics on synaptic signalling (e.g., Forest et al., 2017; Meda et al., 2014; Richiardi et al., 2015; Whitaker et al., 2016; Willemsen et al., 2013). For the first time we were able to demonstrate proof‐of‐concept that dynamic connectivity profiles of individuals with a gene mutation are significantly altered and, crucially, that the extent of this alteration is strongly associated with the expression profile of the gene. Critically, we have demonstrated a valuable method for future work, which should seek to identify the nature of brain network dynamics and their role in the emergence of higher‐level cognitive abilities over development.

## Data Availability

The data that support the findings of this study are available from the corresponding author upon reasonable request.
